# Chemical Vapour Deposition of Graphene for Durable Anticorrosive Coating on Copper

**DOI:** 10.3390/nano10122511

**Published:** 2020-12-14

**Authors:** Dali Ji, Xinyue Wen, Tobias Foller, Yi You, Fei Wang, Rakesh Joshi

**Affiliations:** 1 School of Materials Science and Engineering, University of New South Wales, Sydney 2052, Australia; dali.ji@student.unsw.edu.au (D.J.); xinyue.wen@unsw.edu.au (X.W.); t.foller@student.unsw.edu.au (T.F.); fei.wang3@unsw.edu.au (F.W.); 2 Department of Physics and Astronomy, University of Manchester, Manchester M13 9PL, UK; yi.you@manchester.ac.uk

**Keywords:** graphene, anti-corrosion, chemical vapour deposition, ethanol

## Abstract

Due to the excellent chemical inertness, graphene can be used as an anti-corrosive coating to protect metal surfaces. Here, we report the growth of graphene by using a chemical vapour deposition (CVD) process with ethanol as a carbon source. Surface and structural characterisations of CVD grown films suggest the formation of double-layer graphene. Electrochemical impedance spectroscopy has been used to study the anticorrosion behaviour of the CVD grown graphene layer. The observed corrosion rate of 8.08 × 10^−14^ m/s for graphene-coated copper is 24 times lower than the value for pure copper which shows the potential of graphene as the anticorrosive layer. Furthermore, we observed no significant changes in anticorrosive behaviour of the graphene coated copper samples stored in ambient environment for more than one year.

## 1. Introduction

Corrosion is a naturally occurring phenomenon, which describes the tendency of a material to react with other chemicals resulting in deterioration of stability [1]. A feasible method to reduce the corrosion damage is by coating the metal with a stable and anticorrosive layer. Nevertheless, those traditional protective coatings, including organic layers [2,3,4], polymers coating [5,6], oxide layers [7], and inert metals [8] have their limitations. For example, the high thickness is necessary for traditional anticorrosive layers to ensure protective effects [9,10,11,12]. And some organic or polymers coatings such as epoxy coating can rapidly age under high temperature [13]. Therefore, it is in high demand to solve the corrosion problems by exploiting new chemically inert protective coating materials.

Graphene is a hexagonal two-dimension atomically thin film of carbon. It exhibits many unique properties, including outstanding mechanical properties [14,15,16,17,18] and remarkable chemical inertness in the erosive environment [19,20]. Moreover, graphene also shows exceptional high-temperature stability [21,22]. These properties enable graphene as an ideal candidate for anti-corrosion coating. Additionally, according to Bunch et al., the monolayer graphene sheet is impermeable to any gas molecules, which is essential as an anti-corrosion coating [23]. Chen et al. firstly found that chemical vapour deposition (CVD) graphene grown on Cu and Cu/Ni substrates shows an active resistance against hydrogen peroxide [24]. Kirkland et al. further demonstrated the anticorrosive mechanism for a short time test of graphene coating and reported the impermeability and stability of graphene [25,26]. Schriver et al. and Zhou et al. conducted short term and long-term anticorrosive behaviour of graphene coating and suggested that anticorrosive behaviour is better for the short term. According to the authors, the oxidizing agent can permeate through defects in graphene leading to a faster galvanic corrosion for the long term corrosion mesurements [27,28]. Authors suggested that the permeation of oxidising agent creates a corrosion products between copper and graphene which further accelerates the corrosion rate by exfoliation of graphene [29]. In another works, Ying et al. and Yinghao et al. demonstrated that double layer graphene can efficiently protect the copper from being corroded in the long term. Braeuninger-Weimer et al. have studied the effect of copper crystal orientation on graphene growth and anticorrosive properties. According to the authors, Cu crystal orientations such as (011) exhibiting weak interaction force with graphene cannot prevent the intercalation of corrosive agents, hence resulting into corrosion [30]. However, copper with crystal orientations as Cu(111)and Cu(311) offer relatively strong interfacial coupling with graphene leading to an improved anticorrosive behaviour [30,31].

To improve the coating quality, gas precursors such as methane (CH_4_) have been used in these papers because it provides stable carbon flow [32,33]. However, the average growth temperature of experiments was around 1000 °C, and the growth pressure was high [12,19,26,34]. Here, we report the CVD graphene using ethanol as a carbon source for anticorrosive coating. We observed that the graphene coating prepared by this method could provide considerable corrosion resistance over the large area of the surface.

## 2. Materials and Methods

### 2.1. Growth of Graphene

**Figure 1:**
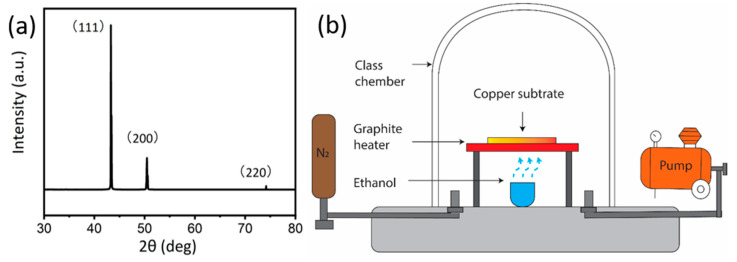
(**a**) The X-ray diffraction (XRD) pattern of Cu substrate showing polycrystalline nature. (**b**) The schematic diagram of the chemical vapour deposition (CVD) system.

In this experiment, we used the polycrystalline copper (A&E Metals Merchants, Sydney Australia, size 2 cm × 1.5 cm × 0.1 mm) as the substrate of graphene coating. All of the substrates were originally sonicated in acetone solution (100%) for 5 min to remove organic residues. After this, the ethanol solution (100%) and deionized water were used to wash samples three times to remove the residual solution followed by heat treatment at 900 °C for 20 min to transform polycrystalline copper into single crystalline Cu(111). The polycrystalline copper substrate after the heat treatment is shown in Figure 1a. Here, it is worth to mention that no pre-treatment such as extra polishing/electroplating has been done on the copper substrate.

The schematic of the CVD system (Microphase Pty Ltd., Manila, Philippines, Maximum temperature 900 °C) is shown in Figure 1, which has been reported in our previous research [35]. The copper substrates were heated by a graphite joule heater which is connected to a direct current temperature controller. An Alumina crucible under the graphite heater was used to load the ethanol precursor. Ethanol has a simple molecular structure, and it is relatively abundant, non-poisonous, and less flammable than traditional gaseous precursor [35,36], hence, it was chosen as our carbon source. The graphene growth was conducted under a stable vacuum of 10 torr (~1.3 kPa). We have used a rotary pump (ULVAC G-20DA) with 100 W 24 L/min (100 V 50 Hz). Prior to the growth, the glass vacuum chamber was purged with N_2_ three times to remove any residual air for growth process. Our experimental conditions allow a heating rate of 18 °C/s and cooling rate of 7 °C/s. To optimize the growth parameters for graphene growth, a set of temperature and growth times were selected (shown in Table 1), and each condition was repeated at least three times to ensure the reproducibility of the results. The infrared thermometer (Model: RS232c output type tmh9) was used for temperature measuring. For clarity, samples are labelled as G-growth temperature-growth time. For example, the sample grown at 750 °C for 5 min is referred to as G-750-5, here G stands for graphene.

### 2.2. Characterization

CVD grown films on the copper surface were thoroughly characterised. Scanning electron microscopy (F.E.I. Nova NanoSEM 230 and NanoSEM 450) was employed to investigate the surface morphology, and Raman spectra were recorded on a Renishaw inVia with 532 nm excitation laser to confirm the crystallinity and quality of graphene. Considering the possible surface inhomogeneity, at least three different spots on the surface were investigated to determine the characteristics of CVD grown films. The presented spectra are representative curves with baseline corrected.

Potentiodynamic polarization and electrochemical impedance spectroscopy (EIS) study was conducted using a Versatile Multi-potentiostat VSP300 with EC-lab software to process the electrochemical data. A glass corrosion cell with the three-electrodes system was used for the electrochemical measurements with a platinum wire as a counter electrode, Ag/AgCl electrode as a reference electrode, and NaCl solution (3.5%) as an electrolyte at room temperature of 23 ± 1 °C. In our measurements, the open circuit potential was monitored for the first 30 min to ensure that the system is operating in high stability. The scan rate of potentiodynamic polarization curves was chosen at 0.5 mV/s in the range −250 mV to 250 mV. EIS tests were conducted by using a sinusoidal potential wave with an amplitude of 10 mV at corrosion potential and the frequencies measuring impedance response between 1 MHz and 10 MHz. All the above tests were repeated at least three times to ensure the reproducibility of the results.

## 3. Results and Discussion

**Figure 2:**
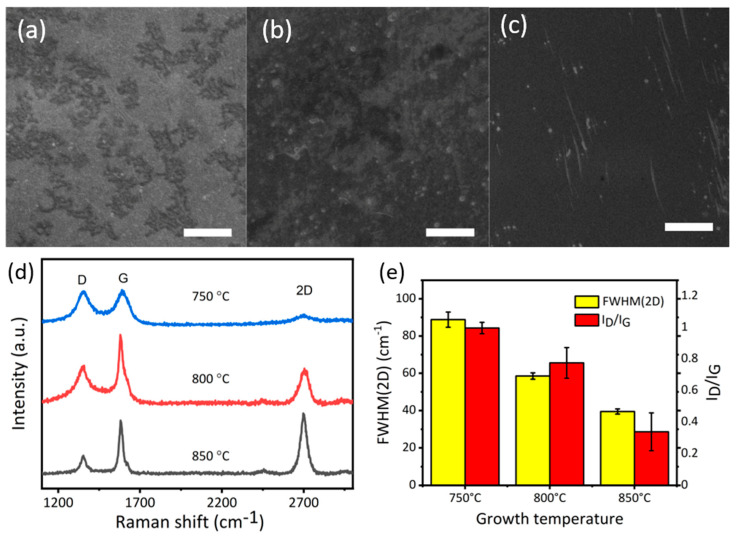
(**a**) Scanning electron microscope (SEM) image of G-750-5; (**b**) SEM image of G-800-5 (**c**) SEM image of G-850-5; (**d**) Raman spectra of samples grown at 750 °C, 800 °C, and 850 °C; (**e**) The full width at half maximum values of 2D peaks (full width at half maximum (FWHM)(2D)) and the ratio of D peak and G peak (I_D_/I_G_); scale bar (**a**–**c**) 3 µm.

As factors such as coverage, layer numbers, and defects can affect the anticorrosive ability of graphene, we optimized the growth parameter before the corrosion test. CVD growth on the copper substrate was studied as a function of growth time and temperature. In our study, we firstly varied the growth temperature by keeping the time constant as 5 min. We analysed the films grown on the substrate using e scanning electron microscopy. Figure 2a shows the typical morphology of the G-750-5. Figure 2d shows the Raman spectra of black areas on the surface. Raman spectra showed a broad D (~1350 cm^−1^) and G peak (~1580 cm^−1^) as well as a very low intensity of the 2D peak (around 2700 cm^−1^) suggesting the formation of graphitic structure [37,38]. Furthermore, the full width at half maximum (FWHM) values of 2D peaks was around 90, and the ratio of D peak and G peak (I_D_/I_G_) is close to 1 (Figure 2e), indicating the disorder [38].

In the second set of experiments, when the temperature was ramped up to 800 °C, the coverage of carbon area increased which is possibly due to the formation of graphene (SEM image, Figure 2b). Raman spectrum indicates an increase in the intensity of G peak, suggesting an increase in the degree of crystallization of carbon (Figure 2d). Another obvious change is the FWHM (2D peak) values for the sample G-850-5 decreasing to ~55 (Figure 2e), which is, however, still higher than the value reported for graphene in the past [38]. This study suggests that the film on the substrate is few (5)-layer graphene with a high ID/IG ratio indicating the presence of defects [37,38]. Furthermore, in comparison to samples G-800-5, the surface of the samples G-850-5 is more uniform with better coverage (Figure 2c). Raman spectra can also demonstrate this well-organized trend. In Figure 2e, the 2D peak of G-850-5 is sharp and symmetrical with FWHM values of it about 39 cm^-1^, suggesting a high level of crystallization and the formation of bilayer graphene [19,38] with low ratio of the intensity of D peak to G peak (I_D_/I_G_); value of about 0.3 [38]. The above study suggested that the graphitic structure is more ordered with the increase in temperature.

**Figure 3:**
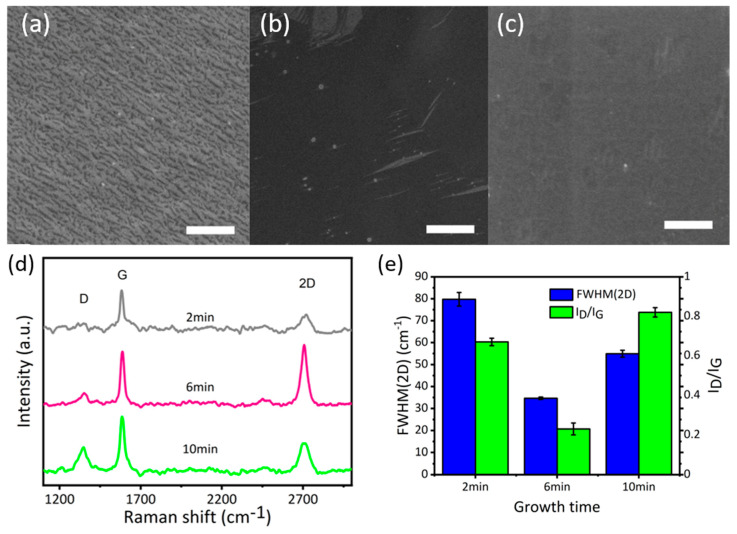
(**a**) SEM image of G-850-2; (**b**) SEM image of G-850-6; (**c**) SEM image of 1G-850-10; (**d**) Raman spectra of graphene samples grown in 2 min, 6 min and 10 min; (**e**) FWHM(2D) and I_D_/I_G_ of graphene samples grown in 2 min, 6 min, and 10 min; scale bar (**a**–**c**) 3 µm.

After determining the optimal temperature of 850 °C for graphene growth, the effect of growth time (2 min, 6 min, and 10 min) on graphene growth has been analysed at 850 °C. Carbon flakes grown at this temperature 2 min were in the shape of small black dots or lines (Figure 3). Raman spectra of this sample presented a low-intensity G peak and a broad 2D peak, which is an indicator of graphite [37,38]. When growth time increases to 6 min (G-850-6 SEM—Figure 3b) the surface is fully covered by the film of double-layer graphene, as illustrated in Raman spectra. It can be seen from the SEM images and Raman spectra that the samples G-850-6 and 850-5 have similar nature, indicating the high stability of the method. When growth time increases to 10 min (sample G-850-10), a degradation in films quality can be observed using Raman spectra analysis (Figure 3d). However, the SEM image showed a fully covered carbon layer. This might be due the fact that the excess alcohol vapours cannot be straightaway removed and allow the formation of excess graphitic layers on substrate with defects [37,38]. To sum up, the optimal growth condition for graphene growth is at 850 °C for 5–6 min, which is more effective to produce bilayer graphene in comparison to the existing methods [29,31,39].

**Figure 4:**
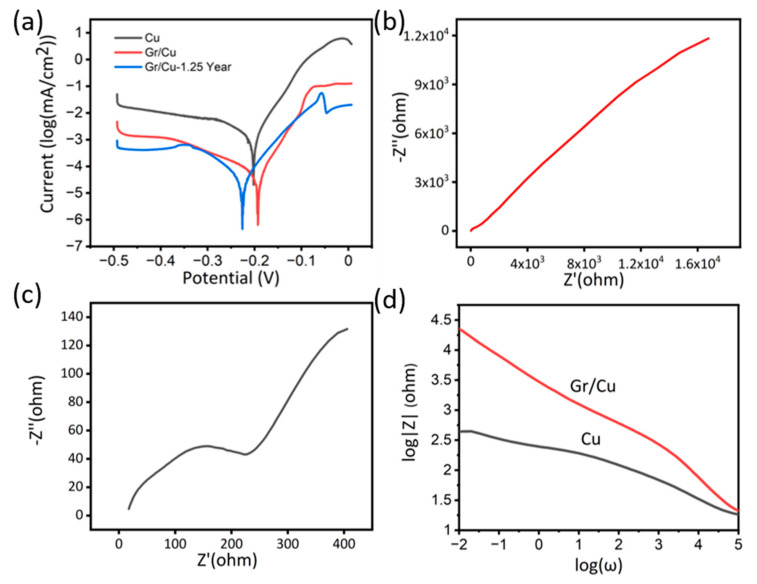
(**a**) Potentiodynamic polarization of the pure Cu, graphene coated sample (Gr/Cu) and graphene-coated sample tested after 1.25 year (Gr/Cu −1.25 Year). (**b**) Nyquist plots of the Gr/Cu and Gr/Cu −1.25 Year. (**c**) Nyquist plots of the Cu. (**d**) Bode magnitude plots of Gr/Cu −1.25 Year, Gr/Cu and Cu sample.

The above study suggests that a temperature of 850 °C and growth time of 6 min is the optimized condition to get good quality graphene films with high coverage. These samples were chosen for anti-corrosion test using an electrochemical method. Figure 4a shows the potentiodynamic polarization measurements data for copper (black curve) and graphene-coated copper (red curve). The data was recorded under high overpotential with electrochemical reaction governed by the Butler–Volmer equation [40]. Thus, a fit (dash line in Figure 4a) to the logarithmic relationship of the current density (I) vs. the electrode potential (V) can provide the redox reaction rate [40]. In the process of corrosion, the anodic dissolution rate of Cu at a specific potential is determined by the anodic current densities stemming from the rate of redox reaction [41].

Consequently, potentiodynamic polarization was applied for the quantitative determination of corrosion rates. From Figure 4a, we can see that the anodic current densities of the Cu samples were nearly one order of magnitude higher than the graphene-coated samples. For the determination of corrosion current, the calculation of Equivalent Weight (*EW*) of copper is required, which can be calculated as below:

(1) $$EW=\;\frac{\;Atomic\;Weight\;of\;Cu}{Valency\;of\;Cu}$$

The calculation for the corrosion rate (*CR*) can be determined by this equation [34,42]:

(2) $$CR=\frac{I_{corr}\times K\times EW}{\rho A}$$

Here, the atomic weight of Cu is 64 g/mol and the valency is +2. The operative area of each sample is 3 cm^2^, and ρ the density of copper substrate is 8.91 g/cm^3^, and the corrosion rate constant K = 3272 mm/(A cm year) [42,43]. From the potentiodynamic polarization curve, the average corrosion current (*I_corr_*) of bare substrates and graphene-coated samples is 1.58 × 10^−3^ mA and 6.51 × 10^−5^ mA, respectively.

After the calculation, the average CR of the bare copper substrate is 1.97 × 10^−13^ m/s. The graphene covered sample mitigates the corrosion, with an average CR of 8.08 × 10^−14^ m/s, which is 24 times lower than the copper corrosion rate. This anti-corrosion effect is higher than the values reported previously [26,34], and with more top surface coverage. This is relatively higher than some traditional coatings such as using benzotriazole which reduces corrosion rate by 10 times [44,45,46,47].

Another electrochemical experiment applied to test the anti-corrosion ability of coating is EIS Nyquist plots are shown in Figure 4b,c to describe the linear relationship between the real impedance (*Z*’) and the imaginary impedance (*Z*’’) [40].

(3) $$Z^{2}={Z^{\prime }}^{2}+{Z^{\prime \prime }}^{2}$$

According to the above equation, when the *Z*’’ tends to be zero, the absolute value of impedance Z is equal to that of *Z*’ which can be approximated to the resistance of sample surface. Consequently, the diameter of the semicircle in the Nyquist plot can represent the ability of electron transfer in the graphene coating or the surface of a bare copper substrate [26]. It is evident from Figure 4b,c that the graphene coating can dramatically increase the impedance of the sample surface and suppress the electron transfer [26,34].

In addition, we analysed the data in the form of bode plots which provides the relationship between impedance Z and frequency ω. In the whole process of ω decreasing, the Z of coated samples exceed that of bare substrates. When the ω is sufficiently small (the y-intercept) and Z mainly results from *Z*’ [26,40], the impedance of the graphene coating sample is much higher compared with the copper substrate. The EIS results are consistent with the polarization results.

Moreover, to study the stability of anticorrosive behaviour, we conducted the corrosion test experiments on samples after 15 months of the first test. The samples were stored in an ambient environment. The potentiodynamic polarization results are shown in Figure 4a. As we can see, there is no significant change in corrosion current on samples tested after 1.25 years, suggesting the highly inert behaviour of our double layer graphene coating as previously observed by Ying et al. [39].

## 4. Conclusions

In this work, graphene films were grown by evaporating ethanol in a CVD system, and we found that the films can serve as anti-corrosive layers. Graphene films grown at a temperature of 850 °C for 5–6 min show excellent anti-corrosion property over significant surface coverage. Our study shows that good quality graphene films can be prepared using readily available ethanol as a carbon source in a CVD process. The potentiodynamic polarization and EIS also separately confirmed that, compared with bare Cu substrates, lower corrosion rate and higher impedance can be achieved by graphene coating.

## Figures and Tables

**Table 1:** Summary of CVD conditions.

Growth Temperature (Growth Time—5 min)	750 °C	800 °C	850 °C
Growth time (growth temperature—850 °C)	2 min	6 min	10 min
